# Low-Dose Dextromethorphan, a NADPH Oxidase Inhibitor, Reduces Blood Pressure and Enhances Vascular Protection in Experimental Hypertension

**DOI:** 10.1371/journal.pone.0046067

**Published:** 2012-09-25

**Authors:** Tao-Cheng Wu, Chih-Yu Chao, Shing-Jong Lin, Jaw-Wen Chen

**Affiliations:** 1 Division of Cardiology, Department of Medicine, Taipei Veterans General Hospital, Taipei, Taiwan, ROC; 2 Cardiovascular Research Center, National Yang-Ming University, Taipei, Taiwan, ROC; 3 Institute of Clinical Medicine, National Yang-Ming University, Taipei, Taiwan, ROC; 4 Department of Medical Research and Education, Taipei Veterans General Hospital, Taipei, Taiwan, ROC; 5 Institute of Pharmacology, National Yang-Ming University, Taipei, Taiwan, ROC; Medical University Innsbruck, Austria

## Abstract

**Background:**

Vascular oxidative stress may be increased with age and aggravate endothelial dysfunction and vascular injury in hypertension. This study aimed to investigate the effects of dextromethorphan (DM), a NADPH oxidase inhibitor, either alone or in combination treatment, on blood pressure (BP) and vascular protection in aged spontaneous hypertensive rats (SHRs).

**Methodology/Principal Findings:**

Eighteen-week-old WKY rats and SHRs were housed for 2 weeks. SHRs were randomly assigned to one of the 12 groups: untreated; DM monotherapy with 1, 5 or 25 mg/kg/day; amlodipine (AM, a calcium channel blocker) monotherapy with 1 or 5 mg/kg/day; and combination therapy of DM 1, 5 or 25 mg/kg/day with AM 1 or 5 mg/kg/day individually for 4 weeks. The *in vitro* effects of DM were also examined. In SHRs, AM monotherapy dose-dependently reduced arterial systolic BP. DM in various doses significantly and similarly reduced arterial systolic BP. Combination of DM with AM gave additive effects on BP reduction. DM, either alone or in combination with AM, improved aortic endothelial function indicated by *ex vivo* acetylcholine-induced relaxation. The combination of low-dose DM with AM gave most significant inhibition on aortic wall thickness in SHRs. Plasma total antioxidant status was significantly increased by all the therapies except for the combination of high-dose DM with high-dose AM. Serum nitrite and nitrate level was significantly reduced by AM but not by DM or the combination of DM with AM. Furthermore, *in vitro* treatment with DM reduced angiotensin II-induced reactive oxygen species and NADPH oxidase activation in human aortic endothelial cells.

**Conclusions/Significance:**

Treatment of DM reduced BP and enhanced vascular protection probably by inhibiting vascular NADPH oxidase in aged hypertensive animals with or without AM treatment. It provides the potential rationale to a novel combination treatment with low-dose DM and AM in clinical hypertension.

## Introduction

It is well known that blood pressure (BP) could be increased with age and hypertension is a public health problem that affects >25% of the adult population worldwide [1], [2]. Hypertension has been identified as the leading risk factor for mortality and ranks as the third-leading cause of disability-adjusted life-years [1], [3]. Despite the availability of numerous antihypertensive agents, current antihypertensive treatment does not always provide sufficient BP control and cardiovascular protection [4]–[6]. The combination therapy with two or more classes of antihypertensive agents is a strategy adopted for improving BP control and cardiovascular protection, which has been suggested in recent guidelines even as an initial therapeutic option [7], [8].

Among the various classes of antihypertensive medications currently available, calcium channel blockers (CCBs) including amlodipine (AM) are one of the most popular first-line treatments including that for aged people [9]–[14]. Though widely prescribed in high-risk and aged patients with multiple risk factors [12]–[16], the use of high-dose CCBs such as AM may be limited due to its relatively less vascular protection in comparison with other antihypertensives [8], [11], [12]. Recent clinical trials suggested that the combination of low-dose CCBs and other medications with particular vascular protective effects might be an attractive alternative strategy especially for elderly hypertension.

It has been shown in both preclinical and clinical studies that during the development of hypertension, the production of superoxide anion (O_2_^−^) derived from NAD(P)H oxidase could be increased with age, which may counteract the enhanced nitric oxide (NO) production derived from inducible NO synthase and generate vasoconstrictor responses on aorta [17]. It is then possible that the inhibition of vascular NAD(P)H oxidase may help to improve BP control as well as vascular protection in the presence of hypertension.

Dextromethorphan (DM) is a dextrorotatory morphinan, which has been widely used as a nonopioid cough suppressant for decades though the exact mechanisms are not clarified [18]. Interestingly, previous studies using animal models of cerebral ischemia and hypoglycemic neural injuries have demonstrated the neuroprotective activity of DM [19]–[24], which might be related to its effects on NADPH oxidase since DM may effectively inhibit the production of reactive oxygen species (ROS) induced by 1-methyl-4-phenyl-1,2,3,6-tetrahydropyridine [25]. However, it was not known whether DM may provide additional cardiovascular protection to hypertension. Accordingly, this study was conducted to test the hypothesis that DM by inhibiting vascular NADPH oxidase may improve BP control and enhance vascular protection in aged hypertensive animals with or without standard antihypertensive treatment such as AM. The *in vitro* endothelial protection effects of DM were also examined. Our findings may provide some novel rationale to the alternative antihypertensive strategy especially for vascular protection in elderly hypertension.

## Materials and Methods

### In vivo study

#### Animals and experimental design

In this study, the 18-week-old male Wistar–Kyoto (WKY) rats were used as control group and the 18-week-old male spontaneous hypertensive rats (SHRs) as the study group. The rats were housed (three per cage) under controlled conditions of temperature, humidity, and light, and had unrestricted access to water. The study protocol was approved by the Animal Experimentation Committee of the Taipei Veterans General Hospital, Taipei, Taiwan, R.O.C. Both WKY rats (*n* = 10) and SHRs (*n* = 120) were housed for 2 weeks and all SHRs were randomly assigned to one of the twelve groups: (1) untreated SHRs (*n* = 10); (2–6) SHRs treated with monotherapy of DM (TTY Biopharm, Taipei, Taiwan) 1, 5 or 25 mg/kg/day and AM 1, 5 mg/kg/day (n = 10 in each group); and (7–12) SHRs treated with combination therapy (combination regimens as DM 1, 5 or 25 mg/kg/day with AM 1, 5 mg/kg/day, n = 10 each group). DM was given to SHRs orally by intragastric gavage as a single dose of 1, 5 or 25 mg/kg/day (DM1, DM5, DM25). AM was administered as single 1, 5 mg/kg/day (AM1, AM5), whereas the DM (1, 5 or 25 mg/kg/day) and AM (1, 5 mg/kg/day) were administered as single combination dose. Control SHRs received 1% solution of methylcellulose (1 ml/kg) by a gavage as a vehicle. Hypotensive drugs were suspended in 1% solution of methylcellulose and administered by a gavage in a 1 ml/kg volume. All compounds were administered for 4 weeks. Arterial BP measurement were carried out after before, the second and the fourth week of drug administration. Blood sampling was done from tail vein before and the fourth week of drug treatment. After completion of drug treatment, all rats were sacrificed and the aortas were collected for vasomotor function assay and immune-staining.

#### Measurement of blood pressure and heart rate

Arterial systolic and diastolic BP were measured in conscious rats with an automatic sphygmomanometer, using tail-cuff method. Before the measurements, the animals were placed inside a warming chamber (about 34°C) for 30 min. The aim of the procedure was to calm the animals and dilate the tail blood vessels. Arterial blood pressure and heart rate were measured in the morning throughout the 4-week periods and the average of five successive recordings was recorded. Changes in BP and heart rate were expressed as the percentage of baseline values.

#### Measurement of vascular reactivity

The experiment was performed according to the previously described method [18]. At the end of the 4-week experimental period, rats were anesthetized with an intraperitoneal injection of sodium pentobarbital. The descending thoracic aorta was excised, freed of fat and connective tissue, cut into rings approximately 2–3 mm in length, and placed in gassed (95% O2 and 5% CO2) Krebs–Henseleit solution with the following composition. The tissues were maintained at 37.8°C under 2-g tension and equilibrated for 1 h before initiating the experimental protocols. During this period, the incubation medium was changed every 15 min. The vascular reactivity was measured in aortic rings with functional endothelium pre-contracted with submaximal concentrations of phenylephrine (10^−5^ mol/l). Endothelium-dependent or endothelium-independent relaxation was evaluated with concentration response curves to acetylcholine (10^−9^ to 10^−5^ mol/l) or sodium nitroprusside (SNP, 10^−10^ to 10^−6^ mol/l), respectively. Relaxation was calculated as the percentage of precontractile vascular tone. The responses of the tissues were recorded using isometric transducers (Kishimoto Medical Instruments, Kyoto, Japan) and recorders (SEKONIC, Tokyo, Japan). The maximal effects for acetylcholine and SNP were expressed as the percentage of relaxation of the precontraction induced by phenylephrine.

#### Measurement of aortic media thickness

Aortas were fixed in 10% buffered formalin and 3-µm sections were prepared from paraffin-embedded tissues. Based on hematoxylin-eosin staining, the media thickness, indicated as the media area of aorta was measured using a computer with a microscopy and software (Image-Pro Plus).

#### Measurement of plasma total anti-oxidative capacity, nitrite/nitrate levels and biomarkers of renin-angiotensin-aldosterone system

Total antioxidant capacity (TAO) was evaluated by enzyme immunoassay (Cayman Chemical Company, An Arbor, MI, U.S.A.). Plasma samples were incubated with nitrate reductase to reduce nitrates to nitrites and final concentration (NOx) was determined by adding Griess reagent to the sample and measuring the absorbance at 540 nm. The NOx concentrations were expressed as µmol/L and calculated using a standard curve of nitrite. The serum levels of renin (Bluegene Biotech CO., LTD.), angiotensin II (Enzo Life Science, United Kindom) and aldosterone (Enzo Life Science, United Kindom) were also measured by enzyme immunoassay.

### In vitro study

#### Cell Culture

Human aortic endothelial cells (HAECs, Cascade Biologics) were grown in Medium 200 (Cascade Biologics) supplemented with low serum growth supplement (Cascade Biologics) in an atmosphere of 95% air and 5% CO_2_ at 37°C in plastic flasks. The final concentrations of the components in Medium 200 contained 2% FBS (GibcoBRL), 1 µg/mL hydrocortisone, 10 ng/mL human epidermal growthfactor, 3 ng/mL human fibroblast growth factor, 10 µg/mL heparin, and 1% antibiotic-antimycotic mixture (GibcoBRL). At confluence, the cells were subcultured at a 1∶3 ratio and used at passage numbers 3 through 8. After incubation with variable concentrations of DM, cell viability was always greater than 90% by MTT assay.

#### The measurement of angiotensin II-induced reactive oxygen species (ROS) production

The effect of DM on angiotensin II-induced ROS production in HAECs was determined with a fluorometric assay using 2′,7′-dichlorofluorescein diacetate (DCFH-DA, Molecular Probes) as a probe for the presence of H_2_O_2_. Confluent HAECs (10^4^ cells/well) in 96-well plates were pretreated with various concentration of DM for 18 hours. After the removal of DM from wells, cells were incubated with 20 µmol/L DCFH-DA for 45 minutes. Angiotensin II was added to the medium for 60 minutes, and the fluorescence intensity (relative fluorescence units) was measured at 485-nm excitation and 530-nm emission using a fluorescence microplate reader [26].

#### The measurement of angiotensin II-induced nicotinamide adenine dinucleotide phosphate (NADPH) oxidase activity

The effects of DM on NADPH oxidase activity of HAECs were measured by lucigenin-enhanced chemiluminescence with the method mentioned by kitada et al. before [27]. In brief, the membrane fraction (15 mg protein) of the cell homogenate after centrifugation was added to glass scintillation vials containing 5 µM lucigenin (Sigma-Aldrich, St Louis, MO, USA) in 1 mL PBS. Superoxide production was measured after adding NADPH (100 µM) into the incubation medium. Nicotinamide adenine dinucleotide phosphate oxidase activity was calculated on the basis of the amount of superoxide produced in the reaction mixture. The chemiluminescence was measured for 15 min and the integrals over this period were expressed as RLU/15 min/mL.

#### The measurement of angiotensin II-induced membrane translocation of p47phox in NADPH oxidase

The HAECs was pretreated with DM and angiotensin II, then the cells were lysed in lysis buffer (50 mM Tris–HCl, pH 7.4, 0.1 mM EDTA, and protease-inhibitor mixture). The membrane fraction of cell lysate was prepared by ultracentrifugation. Western blot analysis was used to determine the membrane fraction levels of NADPH oxidase components membranous p47phox (BD Biosciences Pharmingen, San Diego, CA, USA ).

#### Statistical analysis

Results are expressed as the mean ± SEM. The comparison between pre- and post-treatment was made by Mann-Whitney U test. For statistical evaluation among groups, on-way AVOVA (Kruskall- Wallis test) was sued. A significant P value is less than 0.05.

## Results

### Effects of monotherapy or combination therapy with AM and DM on blood pressure

**Figure 1:**
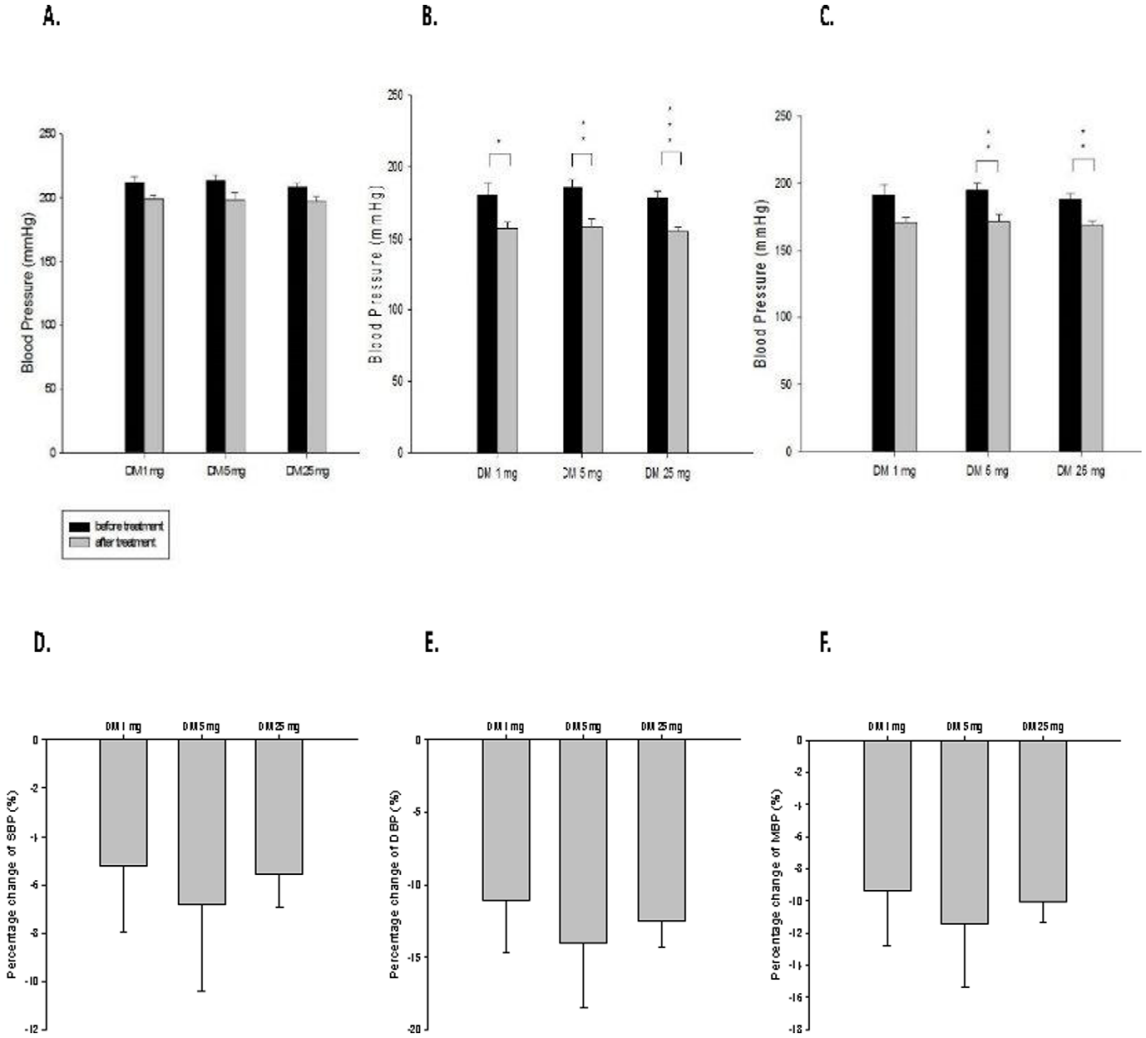
Effect of dextromethorphan (DM) monotherapy on blood pressure of spontaneous hypertension rats. (**A**) systolic blood pressure (SBP) before and after treatment; (**B**) diastolic blood pressure (DBP) before and after treatment; (**C**) mean blood pressure (MBP) before and after treatment; (**D**) percentage changes of SBP by treatment; (**E**) percentage changes of DBP by treatment; (**F**) percentage changes of MBP by treatment, * means P<0.05; ** means P<0.01 ; *** means P<0.001.

The SBP was reduced insignificantly, but DBP was significantly reduced by monotherapy with AM or DM (**Fig. 1** **and** **2**). The BP was similarly reduced by DM treatment in different doses without dose-dependent effects. (**Fig. 1**) The percentage changes of SBP reduction by DM1, DM5, and DM 25 were 5.24±2.74%, 6.79±3.63%, and 5.55±1.36% respectively, that of DBP reduction were 11.11±3.57%, 14.06±4.43%, and 12.54±1.79% respectively, and that of MBP reductionwere 9.37±3.39%, 11.43±3.97%, and 10.02±1.35% respectively. (**Fig. 1**)

**Figure 2:**
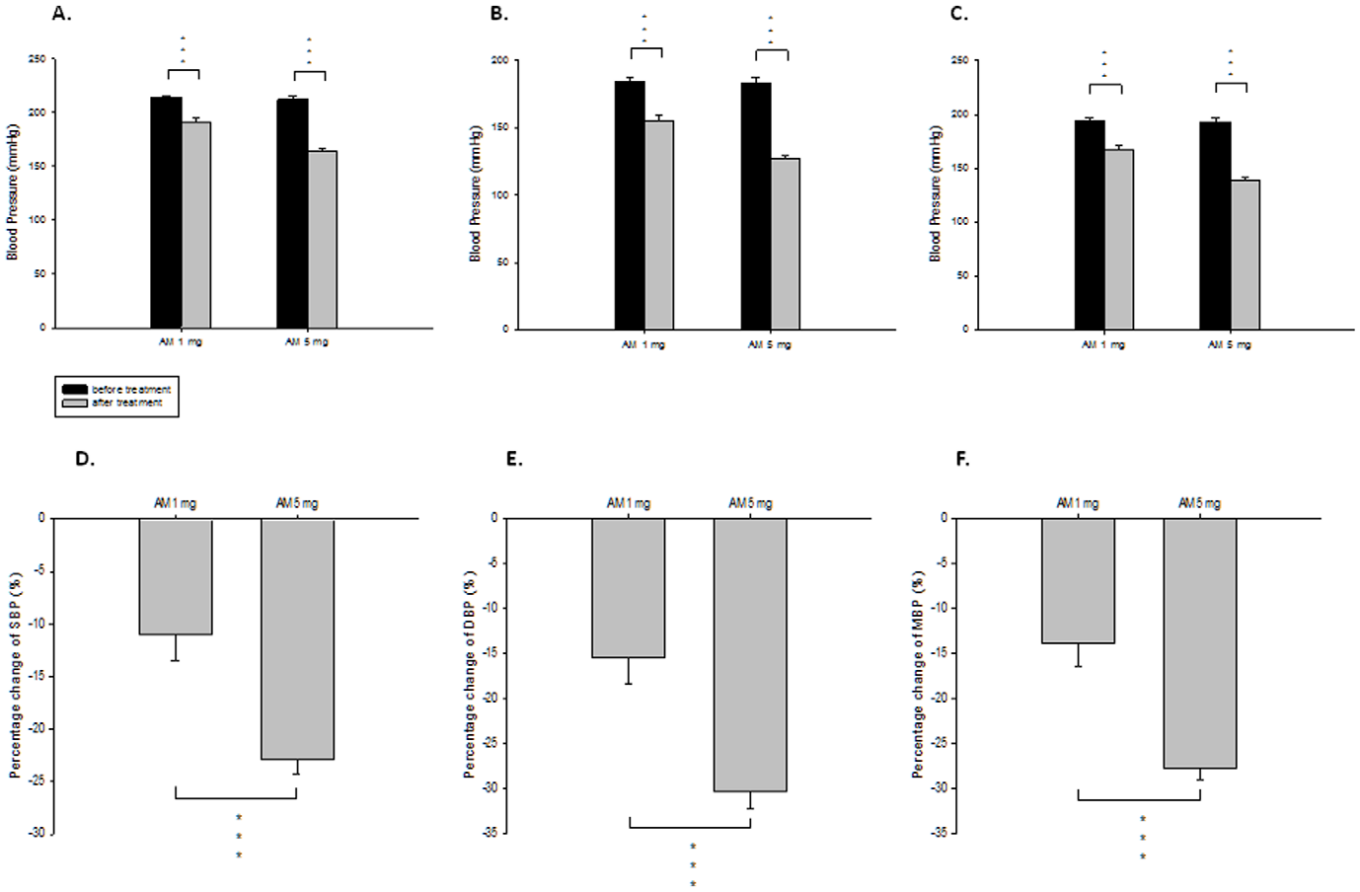
Effect of amlodipine (AM) monotherapy on blood pressure of spontaneous hypertension rats. (**A**) systolic blood pressure (SBP) before and after treatment; (**B**) diastolic blood pressure (DBP) before and after treatment; (**C**) mean blood pressure (MBP) before and after treatment; (**D**) percentage changes of SBP by treatment; (**E**) percentage changes of DBP by treatment; (**F**) percentage changes of MBP by treatment, *** means P<0.001.

On the other hand, BP was reduced dose-dependently by AM treatment. The percentage changes of SBP, DBP and MBP reduction were 11.02±2.44%, 15.50±2.90% and 13.90±2.62% respectively by low dose AM treatment (AM1), and were 22.93±1.33%, 30.38±1.90%, and 27.74±1.23% respectively by high dose AM treatment (AM5). (**Fig. 2**)

**Figure 3:**
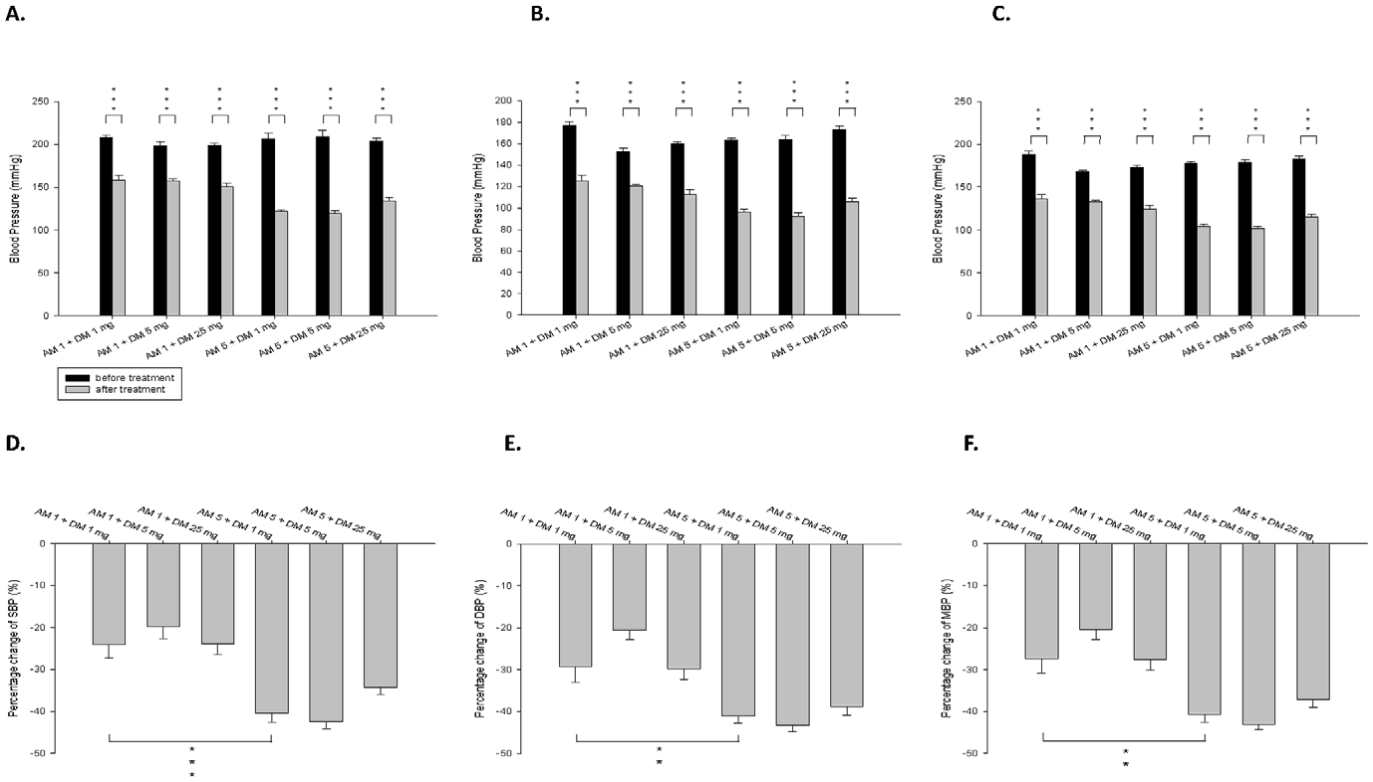
Effects of dextromethorphan (DM) monotherapy or the combination of DM with amlodipine (AM) on blood pressure of spontaneous hypertension rats. SBP: systolic blood pressure; DBP: diastolic blood pressure; MBP: mean blood pressure. * means P<0.05; ** means P<0.01; *** means P<0.001, compared to blood pressure before treatment.

Compared with monotherapy of AM or DM, combination therapy with both AM and DM in various doses had synergistic effects in the reduction of SBP, DBP and MBP. (**Fig. 3**) High dose AM (AM5) in various combinations gave more significant BP reduction than low dose AM (AM 1) in combinations with different doses of DM. However, in each dose of AM, the degree of BP reduction was similar in different combination therapy with various doses of DM. (**Fig. 3D, E, F**) Among all the combinations, high dose of AM with intermediate dose of DM (AM5+DM5) gave the most significant percentage reduction of SBP, DBP and MBP up to 42.44±1.66%, 43.32±1.45%, and 43.12±1.25% respectively. Taken together, the findings showed that while AM montherapy dose-dependently reduced BP, DM monotherapy universally reduced BP without dose-dependent effects. The similar phenomenon was also seen in combination therapy.

### Effects of monotherapy or combination therapy with AM and DM on vascular reactivity

**Figure 4:**
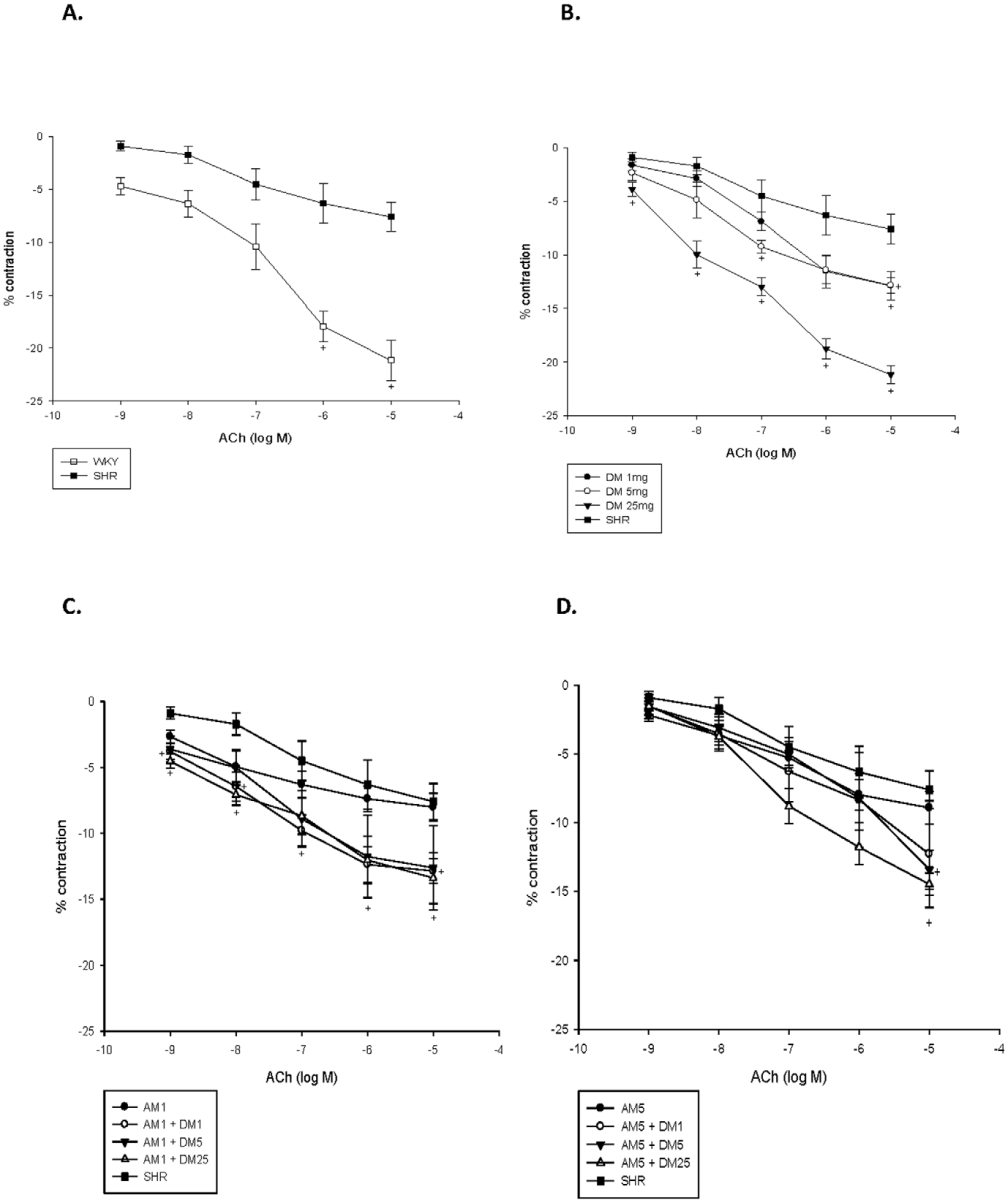
Effect of dextromethorphan (DM) monotherapy or the combination of DM with amlodipine (AM) on acethylcholine-induced vasorelaxation of aortic segments of spontaneous hypertension rats (SHRs). +, P<0.05 compared to SHRs. (**A**) WKY rats versus SHRs; (**B**) SHRs with different dose of M versus SHRs without treatment; (**C**) SHRs with low dose of AM combined with different dose of DM treatment; (**D**)SHRs with high dose of AM combined with different dose of DM treatment.

**Figure 5:**
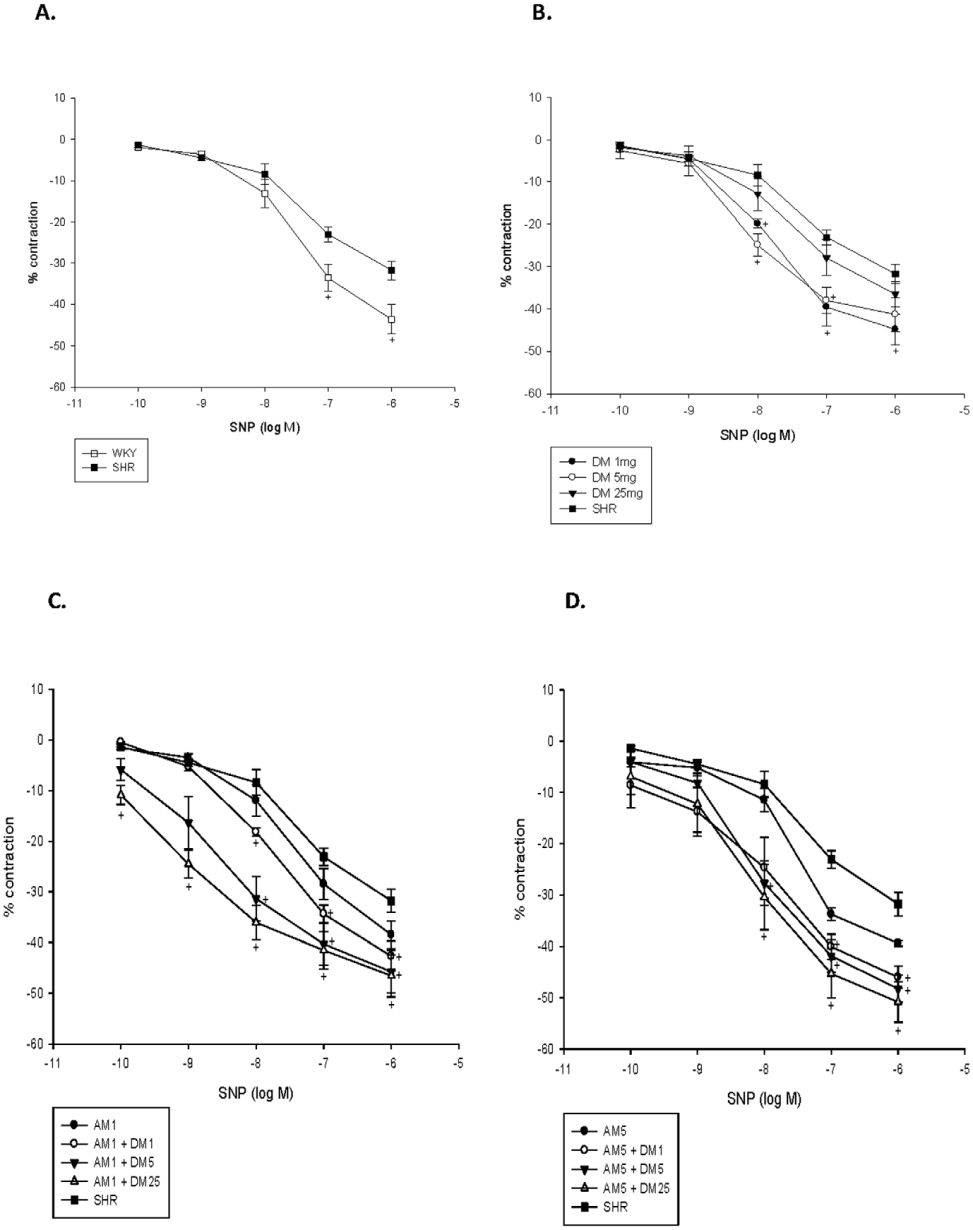
Effect of dextromethorphan (DM) monotherapy or the combination of DM with amlodipine (AM) on SNP-induced vasorelaxation of aortic segments of spontaneous hypertension rats (SHRs). *, P<0.05 compared to SHRs. (**A**) WKY rats versus SHRs; (**B**) SHRs with different dose of DM versus SHRs without treatment; (**C**) SHRs with low dose of AM combined with different dose of DM treatment; (**D**) SHRs with high dose of AM combined with different dose of DM treatment.

Both acetylcholine-induced (endothelium-dependent) vasodilatation and SNP-induced (endothelium-independent) vasodilatation were significantly impaired in the aorta of SHRs as compared with that of WKY rats. (**Fig. 4** **and** **5**) Compared with no treatment, DM monotherapy in different dose increased both endothelium-dependent (**Fig. 4**) and -independent vasodilatation in the aorta of SHRs. (**Fig. 5**) However, such effects were not seen with AM monotherapy in different doses.

Besides, compared with no treatment, combination therapy in different dose significantly increased both endothelial-dependent (**Fig. 4**) and -independent vasodilatation in SHRs. (**Fig. 5**) These findings suggested that while AM monotherapy did not improve vascular function, DM, either alone or in combination with AM, could improve vascular function in experimental hypertension.

### Effects of monotherapy or combination therapy with AM and DM on aortic media thickness/area

**Figure 6:**
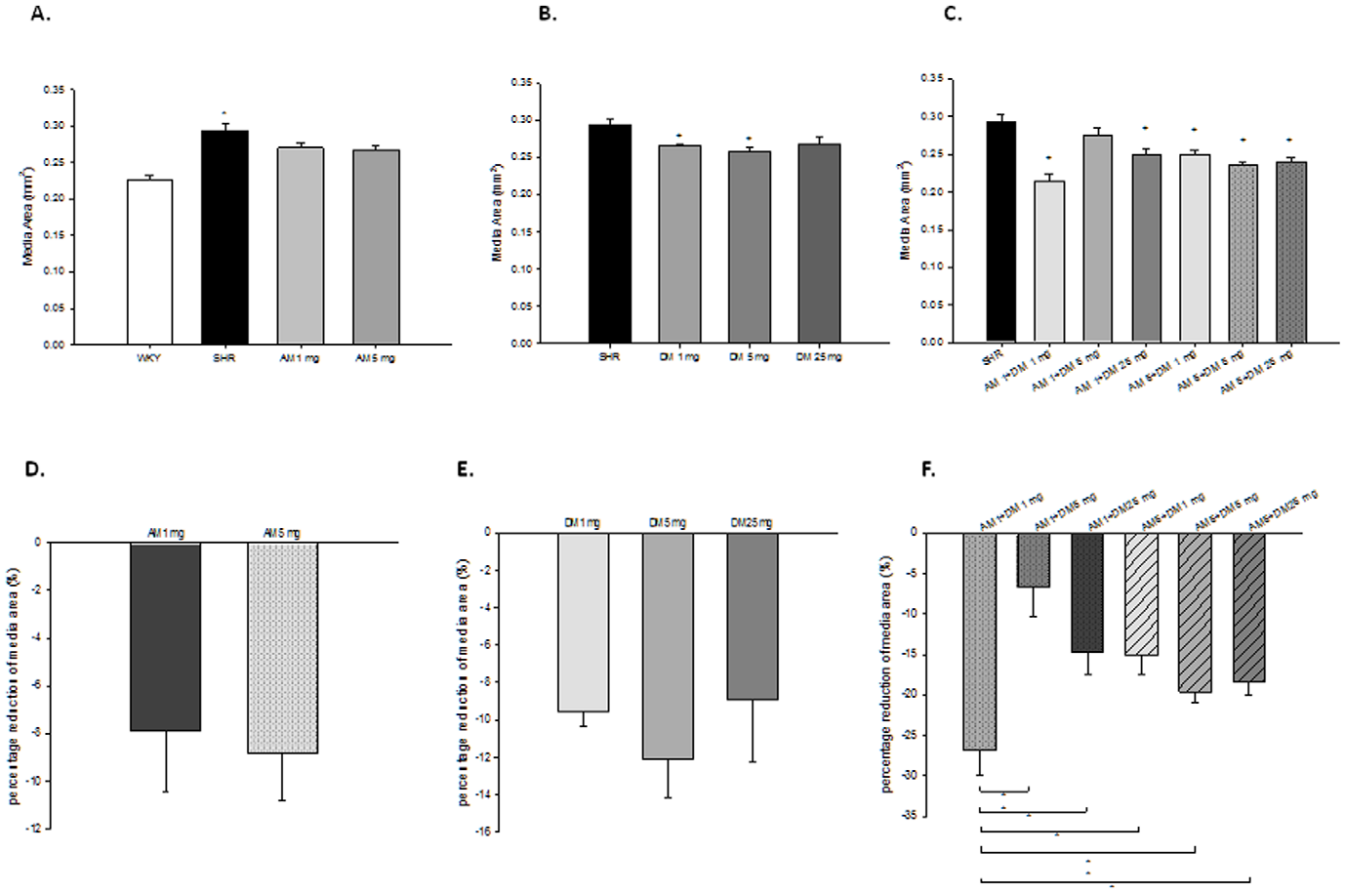
Effect of dextromethorphan (DM) monotherapy or the combination of DM with amlodipine (AM) on vascular media area of aortas of spontaneous hypertension rats (SHRs). (**A**) Effect of AM on the aortic media layer of SHRs. (**B**) Effect of DM on the aortic media layer of SHRs. (**C**) Effect of combination with DM and AM on the aortic media layer of SHRs, + means P<0.05, compared to SHRs. (**D,E,F**) Effect of DM, AM or combination therapy on the percentage change of reduction of media layer of SHRs. + means compared to SHR; * means P<0.05 ; ** means P<0.01.

Media thickness, indicated as media area in the figures, was significantly increased in SHR than in WKY rats. While monotherapy with AM in different doses did not reduce media thickness/area of SHR, low dose and intermediate dose but not high dose of DM significantly reduced aortic media thickness/area of SHRs. (**Fig. 6**) On the other hand, media thickness/area significantly decreased by combination therapy in SHR. Among the different combinations, the combination with low dose of AM (1 mg/kg/day) and DM (1 mg/kg/day) showed the best reduction effect on aortic media thickness/area in SHRs. (**Fig. 6F**) The above findings indicated that DM but not AM monotherapy may have vascular protective effects, and low rather than high dose of DM in combination with AM could have better vascular protective effects.

### Effects of monotherapy or combination therapy of AM and DM on plasma total antioxidant status , nitrite/nitrate level and renin-angiotensin-aldosterone system (RAA)

**Figure 7:**
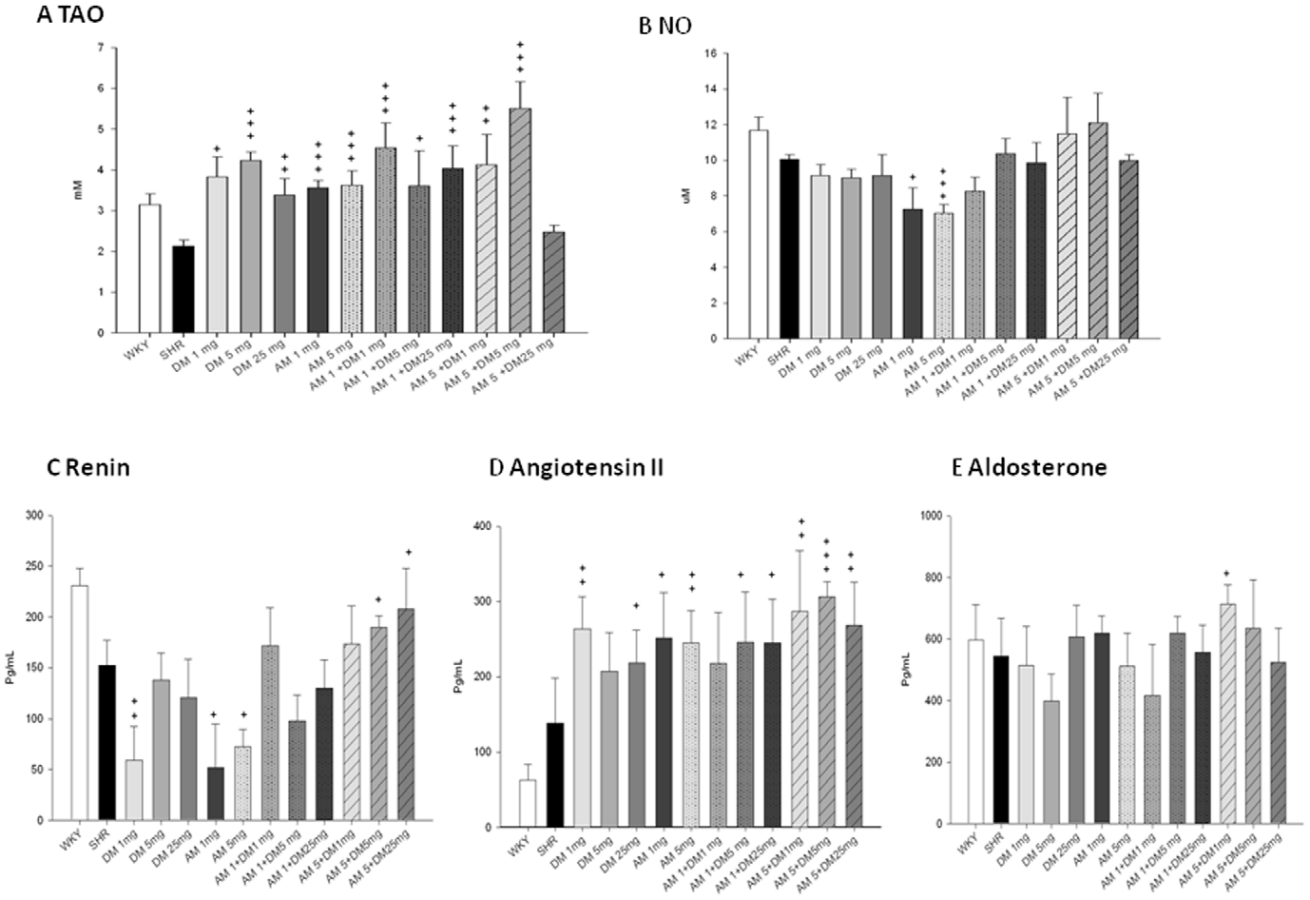
Effect of dextromethorphan (DM) monotherapy or the combination of DM with amlodipine (AM) on the plasma NOx levels. (**A**) and total antioxidant status (**B**) in spontaneous hypertension rats. The serum levels of renin (C) , angiotensin II (D) and aldosterone (E) were also measured. + means P<0.05; ++ means, P<0.01; +++ means P<0.001, compared to plasma NOx levels, total antioxidant capacity and renin-angiotensin system of SHRs.

AM or DM either alone in different doses or in combination with low to intermediate dose significantly increased TAO in SHRs. (**Fig. 7A**) While DM monotherapy did not change plasma NOx level, AM monotherapy, either in low dose or high dose, significantly reduced plasma NOx level as compared with no treatment. On the other hand, combination therapy with different doses of DM and AM did not change plasma NOx level. (**Fig. 7B**) Taken together, the above findings suggested that 1) monotherapy with AM in different dose may increase plasma TAO but reduce plasma NOx level; 2) monotherapy with DM in different dose may increase plasma TAO without alteration of plasma NOx level; 3) combination of DM with AM, except for the high dose combination, may increase plasma TAO without alteration of plasma NOx level; 4) the combination of high dose DM and high dose AM had no effects on either plasma TAO or NOx level.

In RAA system, serum renin level could be reduced by monotherapy with low dose of DM (DM1) or different doses of AM (AM1, 5) but might be increased by high dose combination of DM and AM (DM5+AM5 and DM25+AM5). (**Fig. 7C**) While not changed with the monotherapy of high dose DM (DM5) and the combination of low dose DM and AM (DM1+AM1), serum angiotensin II level was increased by all the other treatments. (**Fig. 7D**) While increased only by the combination of low dose DM and high dose AM (DM1+AM5), serum aldosterone level was not changed by all the other treatments. (**Fig. 7E**) These results showed that the antihypertensive effects of DM and AM might be not related to RAA system.

### *In vitro* effects of dextromethorphan on angiotensin II-induced ROS production and NADPH oxidase activity in HAECs

**Figure 8:**
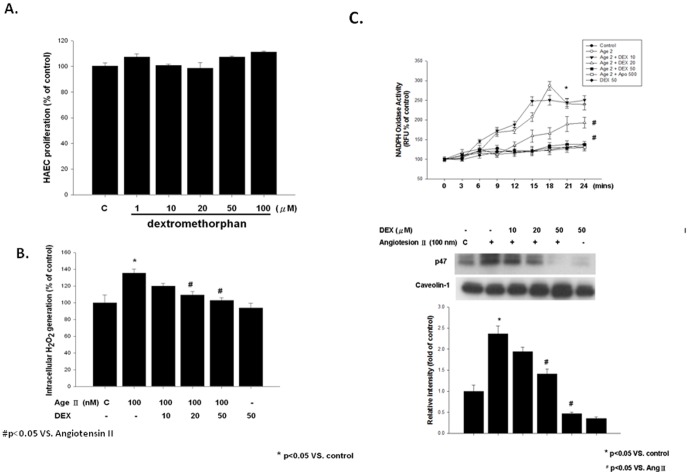
*In vitro* effects of dextromethorphan (Dex) on human aortic endothelial cells (HAECs). (**A**) Effects of various concentrations of Dex on cell viability in MTT assay. Data are expressed as mean±SD of three independent experiments. (**B**) Effect of Dex treatment on angiotensin II-induced superoxide production. Production of superoxide in HAECs was measured by lucigenin-enhanced chemiluminescence. (**C**) Effect of Dex treatment on angiotensin II-induced NADPH oxidase activity in HAECs. Production of NADPH oxidase activity was measured by adding lucigenin and NADPH. (**D**) Effect of Dex treatment on angiotensin II-induced membrane p47phox protein expression in HAECs. Membrane fraction was isolated for western blot analysis of membrane p47phox protein expression. Caveolin was used as an internal control. Bar graphs showed the ratio in percentage of expression intensity of each treatment to that of controls. Data are expressed as mean±SD of three independent experiments. *P<0.05 vs. Control; ^#^P<0.05 compared with the angiotensin II-treated cells.

Exposure to DM (100 µMol/L) for 24 hours did not impair HAECs. Compared with control, angiotensin II (100 nMol/L for 3 hours) significantly increased the ROS production of HAECs, which could be prevented by DM pretreatment. (**Fig. 8A, 8B**) Besides, the NADPH oxidase activity of HAECs was significantly increased by angiotensin II (100 nMol/L for 3 hours), which could be abolished by pretreatment of DM (20 or 50 µMol/L for 18 hours). (**Fig. 8C**)

Furthermore, western blot assay demonstrated that angiotensin II induced p47phox translocation in HEACs. DM pretreatment for 18 hours significantly reduced the membrane translocation of p47phox induced by angiotensin II. (**Fig. 8D**)

## Discussion

The cardinal findings of this study included: 1) while AM montherapy dose-dependently reduced BP, **DM monotherapy** universally reduced BP without dose-dependent effects in SHRs. The similar phenomenon was also seen in combination therapy; 2) while AM monotherapy had no effects on either endothelial-dependent or -independent vasodilatation, **DM**, either as monotherapy or in combination, could improve endothelial-dependent and -independent vasodilatation in the aorta of SHRs; 3) treatment with **DM** but not AM may inhibit vascular hypertrophy; 4) **low-dose** rather than high-dose combination of DM and AM could prevent vascular hypertrophy in SHR; 5) while both could increase plasma TAO, treatment with AM but not **DM** reduced plasma NOx; 6) while both had no effects on plasma NOx level, **low-dose** but not high-dose combination of DM and AM may increase plasma TAO; 7) **DM** dose-dependently reduced angiotensin II-induced ROS production and the activation of NADPH oxidase in human aortic endothelial cells. Accordingly, AM monotherapy may have dose-dependent BP lowering effects without significant vascular protection effects, which might be related to its adverse effects on vascular NO production. On the other hand, DM monotherapy, even in low dose, significantly reduced BP, improved endothelial function, and prevented aortic hypertrophy, which might be related to its *in vivo* as well as *in vitro* antioxidant effects on NADPH oxidase. Furthermore, the combination of low dose DM and AM may exert significant BP lowering and vascular protection effects in experimental hypertension. Our findings may give a rational to future implication of DM, either alone or in combination with AM, on clinical hypertension particularly in those patients with evidence of increased intravascular oxidative stress.

It is suggested that the relationship between ROS and hypertension occurs at the vascular level where oxidative stress induces endothelial dysfunction, vascular inflammation, increased vascular remodeling leading to increased peripheral resistance and elevated BP [28]. It was shown that antioxidant vitamines could reduce BP in some patients with diabetes or hypertension [29]. The increase of antioxidant capacity would also improve endothelial function and hypertension [30]. Previous reports indicated that DM, by directly inhibiting NADPH oxidase activity and consequently decreasing superoxide production, could significantly reduce lipopolysaccharid-induced oxidative stress in microglial cells [25] and in macrophage [31]. However, such effects of DM are not dose-dependent [25], [31]. In the present study, treatment of DM, either alone or in combination therapy, could increase TAO without altering plasma NOx level, suggesting its *in vivo* antioxidant effects in SHRs. There are also no dose-dependent effects of DM on TAO in current study. Furthermore, while low-dose DM therapy significantly reduced BP, higher doses of DM either alone or in combination therapy did not further reduce BP. Taken together, low dose rather than high dose of DM could reduce BP in experimental hypertension, which might be related to the specific *in vivo* effects of DM on vascular NADPH oxidase. Future investigations are required to define the optimal dose of DM before it could be used for clinical hypertension.

Although the physiological and pathophysiological inducers may be complicated and remain poorly defined, intravascular ROS could be theoretically produced by many enzymes including xanthine oxidoreductase, uncouple nitric oxide synthase, and NADPH oxidase [32]–[34]. Besides, decreased antioxidant capacity may also promote oxidative stress and enhance cardiovascular and renal oxidative damage associated with hypertension. [35], [36] It was suggested that in hypertension, increased vascular ROS may reduce NO bioavailability resulting in the loss of its vasoprotective effect [37], and ROS scavengers could attenuate the norepinephrine-induced contraction of rat aorta [38]. In this study, plasma nitrite and nitrate concentrations were measured for systemic NO production [39]. DM, either alone or as combination treatment, improved the attenuated endothelial dependent vasodilation of the aorta in SHR by increasing systemic antioxidant capacity and by upregulating NO bioavailability. Furthermore, DM, either alone or in combination treatment, also improved endothelial-independent vasorelaxation of aortas in response to SNP, suggesting its direct effects on vascular smooth muscle cells. On the other hand, in this study, though significantly reducing BP, AM either in 1 mg or in 5 mg did not alter endothelial-dependent aortic dilatation induced by acetylcholine, suggesting that the effects of AM on BP reduction may be not necessarily associated with the improvement of vascular endothelial function. However, DM, either in 1 mg or in 5 mg, could not only significantly reduce BP but also enhance acetylcholine-induced endothelial-dependent vasodilatation. The endothelial-dependent vasodilatation could be also enhanced while AM was combined with DM. Accordingly, though BP lowering effects may theoretically contribute to vascular protection, it seems more likely that DM could improve endothelial-dependent and -independent vasodilatation and prevent aortic hypertrophy mainly by its direct anti-oxidant effects. Indeed, our *in vitro* findings showed that DM could inhibit the activity/activation of NADPH oxidase and ROS production induced by angiotensin II in HAECs, suggesting the direct effects of DM on vascular endothelium.

Another novel finding of this study is that DM, even in low dose, could significantly decrease BP and further enhance the BP-lowering effects of AM in SHRs. Combination therapy is one of the main strategies in current management of hypertension. Given the fewer side effects, low-dose combination therapy might be more preferred clinically. Current guideline suggests that combination therapy could be anticipated with thiazide, angiotensin converting enzyme inhibitor, angiotensin receptor blocker, CCB or adrenergic β –blocker in various combinations. Our findings show that DM, similar to other first-line antihypertensives, could effectively reduce BP, prevent vascular damage, and improve vascular function in experimental hypertension. Besides, in this study, combination therapy of DM with AM, a CCB, may give the synergetic effects on BP reduction. Interestingly, low dose rather than high dose of DM could give more additional BP reduction top on the AM treatment. The similar effects of low dose vs. high dose of DM on vascular morphology could be also seen in SHRs. Future clinical trials may determine if the combination of low-dose DM and AM could reduce BP and provide efficient vascular protection in clinical hypertension.

In our study, the serum levels of angiotensin II were increased after AM and/or DM treatment. Our findings are similar to that of Konda T and colleagues. They also found that AM could lower BP but increase plasma angiotensin II level in SHR/lzm [40]. On the other hand, Li F and colleagues have reported that losartan (an angiotensin II receptor blockade) treatment could reverse the thickened wall of aorta in the SHR through inhibiting the oxidative stress [41]. It seems that oxidative stress may be more important than angiotensin II itself in the vascular remodeling of the aorta in the SHR. Besides, Bhatia K and colleagues have evaluated the antioxidant capacity in gender difference in SHR after angiotensin II infusion. They found that angiotensin II –induced hypertension was more dependent on the increase of oxidative stress in male than in female SHR [42]. Taking together, one may speculate that although elevating plasma level of angiotensin II, DM could still enhance BP reduction and promote vascular protection via its antioxidant effects.

It was indicated that DM may lead to life threatening complications (arrhythmias, coma, hypertension) when over-dosed. Thus, the serum-concentrations of DM could be critical to the experiment animals. Fiese G and colleagues have evaluated the absorption of DM in rats and showed about 20% absorption of DM from isotonic chloride buffers of Ph 2.0 [43]. It was also shown that at equipotent doses for local anesthesia, DM was safer than bupivacaine (a long-acting local anesthesia) in central nervous system and cardiovascular toxicity. In that study, the highest infusion dose of DM was up to 20 µmoL/kg [44]. Accordingly, it seems that the doses of DM used on rats may be safe in the current study.

## Conclusions

Treatment with DM either alone or in combination with AM could enhance BP reduction and vascular protection, which may be via its NADPH oxidase-related antioxidant effects. The combined treatment of low-dose DM and AM may provide additional BP reduction and vascular protection, which might be an alternative strategy that could be validated especially in aged hypertensive patients at high risk of vascular injury. .
